# The hardest amorphous material

**DOI:** 10.1093/nsr/nwab203

**Published:** 2021-11-13

**Authors:** Yuanzheng Yue

**Affiliations:** Department of Chemistry and Bioscience, Aalborg University, Denmark

It is known that both natural and synthetic solid materials can be classified into three categories: (i) crystalline solids, (ii) amorphous solids and (iii) crystalline-amorphous solids. Crystalline materials are also called ordered materials or crystals, since they feature long-range ordered structure, i.e. the periodic arrangement of lattice points. In contrast, amorphous materials can be referred to as disordered, and include gels, amorphous carbon and silicon, defect-rich solids, and melt-quenched (MQ) glasses, since their microstructure is disordered in the long range (i.e. beyond the scale of ca. 2 nm). It is important to note that amorphous material differs from glass in the following aspects. First, MQ glass is an amorphous material, but the latter is not necessarily glass. Second, MQ glass exhibits a glass–liquid transition upon heating, whereas a non-glassy amorphous material does not show such a transition. Third, the medium-range (i.e. ca. 0.8–2 nm) structure in MQ glass can be either ordered or disordered, depending on the chemical composition of the MQ glass. The short-range structure in MQ glass was generally believed to be ordered until MQ metal-organic framework glass was found to exhibit a high degree of short-range disorder [1]. The term ‘glassy carbon’ often encountered in literature is not an accurate expression, since ‘glassy carbon’ does not exhibit glass transition during cooling or heating. In this sense, ‘amorphous carbon’ is a more appropriate term than ‘glassy carbon’.

Compared to crystalline materials, amorphous materials exhibit unique properties, e.g. higher optical and photonic performances, and can be chemically tuned and easily shaped. But on the other hand, amorphous materials, particularly MQ glasses, are more brittle, weaker (in terms of mechanical strength) and softer than their counterparts—crystalline materials. The drawbacks of amorphous materials arise from their lower atomic packing density, higher potential energy (or lower cohesive energy) and easier initiation and propagation of surface flaws, thus limiting their application. This is why scientists have been continuously attempting to develop superior amorphous materials in mechanical properties [2–5].

**Figure 1.**
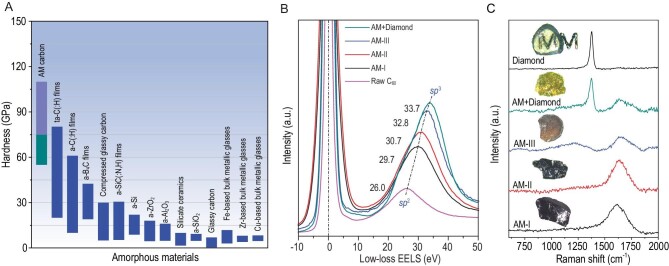
(A) Hardness of AM carbon materials compared with other known amorphous materials. (B) Low-loss EELS data where the shift of the position of the plasmon peak indicates an increase of *sp*^3^ bond fraction. (C) UV Raman spectra of AM-I, AM-II, AM-III, AM+Diamond composite and diamond. Insets: optical photographs of the synthesized samples. Reproduced with permission from ref. [5].

Inspiringly, Zhang *et al*. (i.e. Zhao and Tian's research group) recently made a critical breakthrough in developing superhard and superstrong amorphous materials [5]. They created a new phase of amorphous carbon (AM carbon), namely, AM-III, via relaxation of compressed fullerene C_60_, under a pressure of 25 GPa and a heat-treatment temperature of ∼1200°C. It is amazing that this new phase of amorphous carbon exhibits a Vickers hardness value of ∼113 GPa, which is more than 10 times higher than that of silicate glasses, metallic glasses and other types of amorphous materials (Fig. 1A). It is also higher than that of other types of amorphous carbon. As such, AM-III is the hardest amorphous material known today, and can even scratch crystalline diamond. AM-III displays a compressive strength value of ∼70 GPa, which is comparable to that of diamond. Therefore, it can be stated that AM-III is the champion of amorphous materials in terms of mechanical properties. These outstanding mechanical properties have been ascribed to AM-III’s unique structural features, e.g. a high fraction of *sp*^3^ bonds, as shown in Fig. 1B [5]. A striking change in the short-range order was observed in AM-III, which involves the aromatic ring opening and short chain formation (as confirmed by the Raman spectra in Fig. 1C). Such structural change is accompanied by the interlinking of the structural units via *sp*^3^ bonds. Thus, one can infer that the interlinking increases the number of topological constraints of the structural network, thereby giving rise to the enormous enhancement of both the hardness and compressive strength of AM-III. Furthermore, the new forms of AM carbon developed by Zhang *et al.* [5] are excellent semiconductors with relatively narrow bandgaps (1.5–2.2 eV), and thus they have great potential for photoelectric application.

Another groundbreaking finding achieved by Zhang *et al.* [5] is that polyamorphism occurs in carbon under certain temperature-pressure conditions. To be specific, the low-density-phase AM-I transforms into high-density-phase AM-II, and then into even-higher-density-phase AM-III with increasing temperature under a constant pressure (∼25 GPa). Polyamorphism describes a scenario wherein a substance contains different amorphous phases with the same chemical composition but different thermodynamic states such as density and entropy. The Raman spectra and sample color change shown in Fig. 1C confirm that the structural transition from AM-II to AM-III with temperature takes place in a discontinuous fashion. This transition behavior is similar to that of many other condensed matters such as ice, silicon and ZIF-4 (a metal-organic framework), where a distinct, discontinuous (i.e. first-order) transition occurs between two amorphous phases with different densities. However, the thermodynamic evolution during the polyamorphic transition in carbon still needs to be explored. Polyamorphism remains one of the most important but challenging problems in amorphous materials science. As a critical step, Zhang *et al.* provided new insight into the structural origin of polyamorphic transitions by detecting local structural changes during such transitions [5]. Their discoveries will greatly benefit the development of superhard, superstrong and semiconducting amorphous materials.

***Conflict of interest statement*.** None declared.
